# Identification and Classification of *Coix seed* Storage Years Based on Hyperspectral Imaging Technology Combined with Deep Learning

**DOI:** 10.3390/foods13030498

**Published:** 2024-02-04

**Authors:** Ruibin Bai, Junhui Zhou, Siman Wang, Yue Zhang, Tiegui Nan, Bin Yang, Chu Zhang, Jian Yang

**Affiliations:** 1 State Key Laboratory for Quality Ensurance and Sustainable Use of Dao-di Herbs, National Resource Center for Chinese Materia Medica, China Academy of Chinese Medical Sciences, Beijing 100700, China; bairuibin2022@163.com (R.B.); jh-zhou@nrc.ac.cn (J.Z.); wsm8192023@163.com (S.W.); zhangyuegugude@163.com (Y.Z.); nantiegui@163.com (T.N.); ybinmm@126.com (B.Y.); 2 School of Information Engineering, Huzhou University, Huzhou 313000, China

**Keywords:** food authenticity, *Coix seed*, hyperspectral imaging, classification and recognition, machine learning

## Abstract

Developing a fast and non-destructive methodology to identify the storage years of *Coix seed* is important in safeguarding consumer well-being. This study employed the utilization of hyperspectral imaging (HSI) in conjunction with conventional machine learning techniques such as support vector machines (SVM), k-nearest neighbors (KNN), random forest (RF), extreme gradient boosting (XGBoost), as well as the deep learning method of residual neural network (ResNet), to establish identification models for *Coix seed* samples from different storage years. Under the fusion-based modeling approach, the model’s classification accuracy surpasses that of visible to near infrared (VNIR) and short-wave infrared (SWIR) spectral modeling individually. The classification accuracy of the ResNet model and SVM exceeds that of other conventional machine learning models (KNN, RF, and XGBoost). Redundant variables were further diminished through competitive adaptive reweighted sampling feature wavelength screening, which had less impact on the model’s accuracy. Upon validating the model’s performance using an external validation set, the ResNet model yielded more satisfactory outcomes, exhibiting recognition accuracy exceeding 85%. In conclusion, the comprehensive results demonstrate that the integration of deep learning with HSI techniques effectively distinguishes *Coix seed* samples from different storage years.

## 1. Introduction

*Coix seed*, also known as adlay, Job’s tears, or adlay millet, is the mature kernel of *Coix lacryma-jobi* L. var. ma-yuen (Roman.) Stapf. It has long been used as a health food and medicinal product in East and Southeast Asia [1]. *Coix seed* is rich in proteins, polysaccharides, lipids, polyphenols, and phytosterols and other medicinal substances, which have a variety of physiological and pharmacological effects, such as spleen strengthening, dampness eliminating, anticancer, antioxidant, anti-inflammatory, lipid lowering, and immunomodulating [2]. Therefore, *Coix seed* has been developed and utilized in a wide array of commodities, making it more valuable than other conventional grain commodities [3].

Quality attributes determine the commercial values of *Coix seed*. During storage, *Coix seed* is prone to aging, leading to changes in its starch, protein, and lipid content, which in turn affect its texture, color, and flavor [4]. Additionally, during storage, *Coix seed* may be susceptible to contamination by mycotoxins, toxic secondary metabolites produced by toxic fungi, such as aflatoxins and zearalenone [5]. Therefore, consuming aged *Coix seed* not only impacts the sensory experience but also poses serious health risks to consumers. However, in the market, particularly on online selling platforms, it is common for some merchants to label aged *Coix seed* with a label of fresh seed on its packaging. It was difficult for consumers to distinguish the aged and fresh *Coix seed* with the naked eye due to the similar appearance. Therefore, an effective and rapid method to determine the storage years of *Coix seed* should be developed to protect the interests of consumers.

Traditional methods for identifying storage years of grain include manual observation and chemical detection. However, manual observation methods have the disadvantage of being highly subjective and poorly reproducible [6]. The disadvantages of chemical detection methods are that they are destructive detection techniques, costly to detect, particularly time-consuming, and cumbersome to operate [6,7]. In addition, large-scale detection of the quality attributes of single *Coix seed* is quite difficult for these techniques. Therefore, fast, accurate, non-destructive, and high-throughput detection techniques at the single seed level can provide guidance for the consumption of *Coix seed*.

Hyperspectral imaging (HSI) technology is a non-invasive, non-contact, and rapid detection technology that provides information on the spatial distribution of molecular vibrations in scanned samples [8]. HSI technology has been widely used in the food industry [9]. Recent studies have also shown its potential for single-kernel measurement [10,11]. However, the hyperspectral images consist of numerous contiguous waveband images captured for each pixel of the object, resembling a voluminous cube. This abundance of redundant information poses a challenge in data analysis [12]. The processing of HSI data commonly involves the integration of machine learning techniques, which enable the exploration of the inherent information within the data [7]. An appropriate machine learning approach can proficiently handle the pertinent information contained within HSI data. Through a comprehensive literature review, conventional machine learning methods such as K-nearest neighbor (KNN), random forest (RF), and support vector machine (SVM) integrated with HSI technology have been extensively employed for diverse applications such as variety identification [13], origin traceability [14], and content prediction [15]. The majority of the hyperparameters in these conventional algorithms were manually determined based on prior experience, offering the benefits of a straightforward model structure and low computational cost [16]. Nevertheless, in cases where the sample space is limited or the class separation is not distinct, the model’s generalization capability and stability cannot be assured [17].

Deep learning is a representation learning-based machine learning technique that autonomously learns and uncovers the discriminative features necessary for classification through the hierarchical processing of multiple layers of input data [8]. By employing convolution calculations and fully connected (FC) layers, a convolutional neural networks (CNNs) combine band processing and pattern recognition, thereby diminishing the need for manual intervention and effectively enhancing classification efficiency [18]. CNNs have become an ideal method for analyzing and processing hyperspectral data [19]. Cai et al. captured the spectral information of *Radix Paeoniae Alba* by HSI and fed it to a CNN to distinguish its geographical region [14]. Kong et al. developed a rapid detection method HSI combined with a CNN for the qualitative and quantitative identification of marine fishmeal adulteration [20]. However, as the depth of the network increases, CNNs may encounter the issue of gradient vanishing [21]. A modified CNN structure called ResNet, proposed by He et al. [22], introduces a shortcut mechanism where residual modules are connected through shortcut connections. By employing residual learning, ResNet effectively addresses the degradation problem caused by increasing network depth.

In this study, we mainly attempt to identify the storage years of the *Coix seed* using HSI technology with ResNet algorithms. The specific aims of this study were (1) to collect the visible to near infrared (VNIR) and short-wave infrared (SWIR) HSI data of single *Coix seed* from different storage years; (2) to compare the feasibility of conventional typical machine learning (ML) methods in identifying *Coix seed* storage years; (3) to utilize ResNet models to establish *Coix seed* storage year classification models based on spectra images; (4) to evaluate the performance of the model using external validation sets. The results of this study show that the combination of HSI and ML techniques can successfully determine the storage years of *Coix seed*. This approach provides a fast and non-destructive means of identifying the freshness of *Coix seed* samples.

## 2. Materials and Methods

### 2.1. Sample Preparation

Over a span of three consecutive years, namely 2019, 2020, and 2021, samples of *Coix seeds* were collected from two locations: Xingren City in Guizhou Province and Chuxiong City in Yunnan Province. Each year, two batches of samples were procured from each location, culminating in a grand total of 12 batches (2 × 2 × 3) in aggregate. In the process of sample preparation, a total of 300 *Coix seed* samples, selected at random and devoid of any surface damage, were extracted from each batch. This resulted in a grand total of 3600 (300 × 12) samples that were subsequently employed to generate HSI data. The division of the dataset into training and test sets was conducted randomly, adhering to a ratio of 7:3. In the specific context of the *Coix seed* samples collected from both Yunnan and Guizhou regions, this resulted in 1260 samples allocated to the training set and 540 samples assigned to the test set for each respective region. To build classification models, the class labels of samples from 2019, 2020, and 2021 were set as 0, 1, and 2. Furthermore, an external validation set was established to validate the accuracy of the model in identifying the storage years of the *Coix seed*. This validation set comprised 16 groups, each comprising 96 *Coix seed* grains, with 32 grains in each year.

### 2.2. Hyperspectral Imaging System

**Figure 1:**
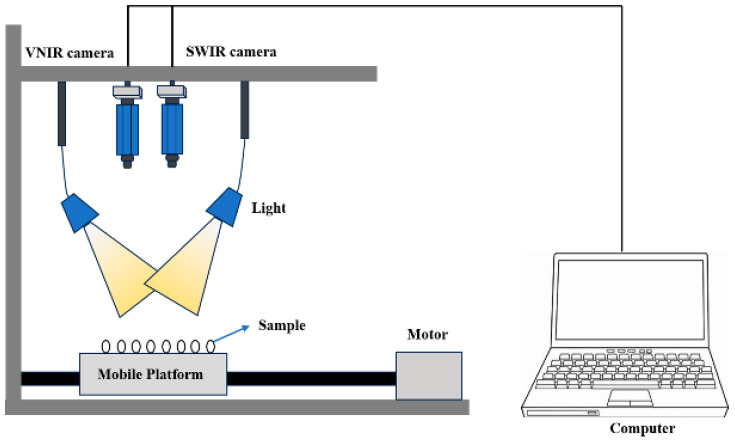
Hyperspectral imaging system.

Data collection was conducted using a visible and short-wave/long-wave near-infrared hyperspectral imaging system (HySpex VNIR-1800/HySpex SWIR 384; Norsk Elektro Optikk, Oslo, Norway). As depicted in Figure 1, the primary components of the system include two lenses covering wavelengths ranging from 410 to 990 nm (VNIR) and 950 to 2500 nm (SWIR), two 150 W halogen lamps (H-LAM; Norsk Elektro Optikk, Oslo, Norway) as the light source, a conveyor belt for sample delivery, and a computer for data collection. The two lamps were set with the incident angle of the light source of 45◦. The exposure time for the VNIR and SWIR lenses was set at 0.0035 s and 0.0045 s, respectively. The distance between the samples and the lens was 32 cm, while the conveyor belt’s moving speed was 2.5 mm/s.

In order to mitigate the influence of instruments and the environment on the sample data, the original spectral data were corrected using software that is compatible with the spectrometer used (HySpex RAD; Norsk Elektro Optikk, Oslo, Norway). Prior to subsequent data analysis, a black–white calibration was performed to eliminate noise caused by variations in the particle shape and dark current in the camera [23]. To handle the black–white calibration, black and white reference images were acquired based on previous research [24]. The black and white correction was carried out using the following formula:

$$R=\frac{R_{0}-{IM}_{dark}}{{IM}_{white}-{IM}_{dark}}$$

where R was the corrected hyperspectral image; R_0_ was the hyperspectral image before correction; IM*_dark_* and IM*_white_* were the black and white reference images, respectively. The region of interest (ROI) was manually extracted using the ENVI 5.3 software (Research Systems Inc., Boulder, CO, USA). Each subsample represented the average value of a specific ROI.

### 2.3. Preprocessing and Feature Wavelength Screening

The presence of noise in the extracted raw spectral data information may hinder subsequent data analysis due to the influence of factors such as test samples, measurement environment, and instrument noise [25]. Hence, it was imperative to employ suitable methods for preprocessing the raw spectral data. In this study, several preprocessing methods were employed, including multiple scatter correction (MSC), standard normal variate (SNV), Savitzky–Golay (SG) smoothing, as well as first and second derivative transformations, to preprocess the raw spectral data.

HSI data exhibit high dimensionality, collinearity, and redundancy. To address this issue and simplify the model, it is crucial to eliminate collinear and irrelevant spectral data from the complete spectral dataset, thereby reducing its dimensionality [26,27]. The competitive adaptive reweighted sampling (CARS) represents a variable selection approach founded on adaptive reweighted sampling, effectively extracting pertinent variables associated with the object of interest [28]. The successive projections algorithm (SPA) serves as a forward variable selection method commonly employed to eliminate the collinear variables within variable combinations [29,30]. Subsequently, the characteristic wavelengths identified by the CARS and SPA methods were utilized to construct a streamlined model.

### 2.4. Conventional Machine Learning Methods

K-nearest neighbor (KNN) is a classification technique used to assign unmarked specimens to categories based on their similarity to labeled specimens [31]. When dealing with unknown samples, the KNN algorithm calculates the distance between the sample and labeled sample of known category. The sample’s class is then determined by considering the votes of its K nearest neighbors. An important consideration in implementing KNN is determining the optimal value for K. Typically, different values of K are tested, and the value that results in the best classification performance is chosen [30].

Random forest (RF) is an ensemble learning algorithm that is based on decision trees and is used for classification or regression tasks [32]. The random forest algorithm combines the outcomes of multiple decision trees to make predictions. One of the key features of random forest is the use of random sampling of training data and random selection of features, which helps to mitigate overfitting. When constructing each decision tree, random forest randomly selects a subset of data from the training set and performs random feature selection. This inherent randomness in the algorithm allows it to effectively address the issue of overfitting and improve the model’s ability to generalize well to unseen data. The accuracy of the results in random forest models is influenced by the number of decision trees (ntree) and the number of randomly selected attributes for splitting (mtry) [33].

Support vector machine (SVM) is a supervised machine learning method that is known for its strong generalization ability, based on the principles of statistical learning theory [34]. SVM has been widely used as an efficient and reliable approach for predicting hyperspectral characteristics. In cases where the data are linearly separable, SVM constructs an optimal discriminative hyperplane in the original feature space to separate samples belonging to different categories. The optimal hyperplane is determined by computing the geometric distances of the samples to the hyperplane and optimizing it to maximize the sum of these distances between the two classes. For situations where the data are not linearly separable, SVM employs a non-linear mapping algorithm to transform the samples from the low-dimensional input space to a higher-dimensional feature space, making them linearly separable. The selection of an appropriate kernel function plays a crucial role in this mapping process, and choosing the optimal kernel function is of great significance [35].

Extreme gradient boosting (XGBoost) is a powerful machine learning algorithm that offers parallel tree boosting, enabling fast and accurate solutions to various data analysis problems [36]. XGBoost is an enhancement of the gradient boosting decision tree (GBDT) algorithm. GBDT utilizes the regression of classification and regression trees (CART) [37]. It starts by training a CART tree and obtaining an output, and then the subsequent trees are trained based on the gradient (e.g., residual error) of the previous tree. In each iteration, a weak classifier is trained to minimize the loss, and the final output of the GBDT model is calculated as the weighted sum of the outputs of all trees. XGBoost builds upon the GBDT framework but introduces a new loss function that enhances convergence and improves performance. This new loss function helps accelerate the learning process of the model. While CART is commonly used as the basic model in GBDT, XGBoost allows for the use of other classifiers as the base model [38]. This flexibility enables XGBoost to handle a wider range of data analysis tasks.

### 2.5. ResNet Model

**Figure 2:**
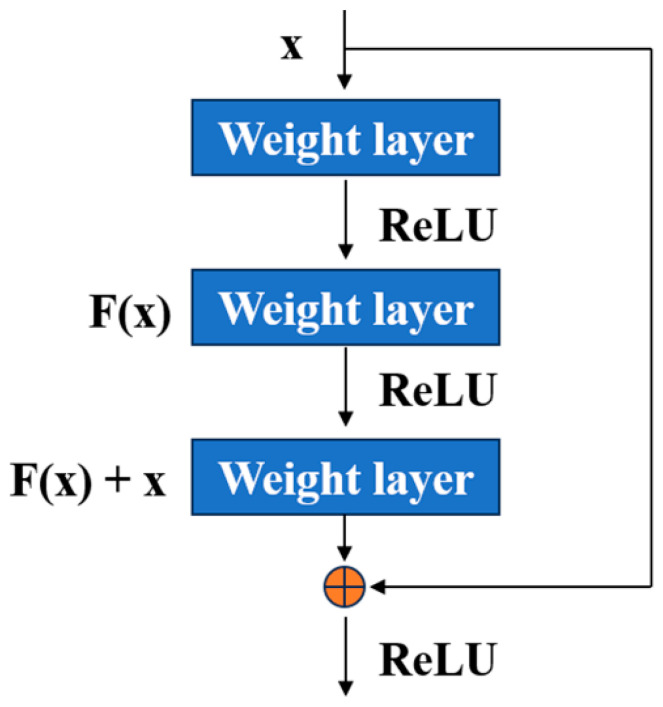
The residual learning block [22].

In this study, the ResNet (residual network) architecture was employed to identify the storage years of *Coix seed*. Figure 2 depicts the structure of the basic bottleneck building blocks that form the ResNet model. The key concept in ResNet is the residual building block, which can be mathematically expressed as *y* = *F*(*x*) + *x*. In this equation, *F*(*x*) represents the residual function, while *x* and *y* denote the input and output parameters of the residual function, respectively. Notably, the output *y* serves as the input *x* for the next residual block in the network.

The ResNet model utilized in this investigation comprises a total of 50 layers, encompassing 49 convolutional layers and 1 fully connected layer. A detailed depiction of the ResNet network’s overall architecture can be found in Table 1, providing a comprehensive overview of its structural composition. The 49 convolutional layers can be classified into five distinct segments. The initial segment involves input preprocessing and encompasses a solitary convolutional layer. The ensuing four segments consist of bottleneck building blocks [39]. Each bottleneck building block is comprised of multiple convolutional layers, batch normalizations, rectified linear unit (ReLU) activation functions, and a shortcut connection [40]. The inclusion of the shortcut connection enables the network to circumvent one or more layers; thereby, facilitating the smooth flow of gradients during training and effectively mitigating the vanishing gradient problem.

### 2.6. Data Analysis and Model Evaluation

The accuracy, precision, specificity, and sensitivity of the model were calculated to evaluate the reliability and stability of the model, based on the following formula:

$$Accuracy\left(\%\right)=\frac{TP+TN}{TP+FP+FN+TN}\times 100\%$$

$$Precision\left(\%\right)=\frac{TP}{TP+FP}\times 100\%$$

$$Specificity\left(\%\right)=\frac{TN}{TN+FP}\times 100\%$$

$$Sensitivity\left(\%\right)=\frac{TP}{TP+FN}\times 100\%$$

where *TP* is true positive; *TN* is true negative; *FP* is false positive; *FN* is false negative. For each index, a higher value represents the better performance of the corresponding model.

The HSI data processing and classification model development were conducted utilizing Python (version 3.9.7 64 bit) in conjunction with PyCharm Community (version 2023.1.2) on the Windows 10 platform. The conventional machine learning algorithms were implemented using scikit-learn (version 1.3.0). The ResNet models were developed utilizing DL Pytorch (version 2.0.1). All data analysis procedures were executed on a computer equipped with 64 GB of RAM, an INTEL i9-12900 K CPU, and an NVIDIA GEFORCE RTX 4080 GPU with CUDA (version 11.8).

## 3. Results

### 3.1. Spectral Profile of Coix Seed Samples from Different Storage Years

**Figure 3:**
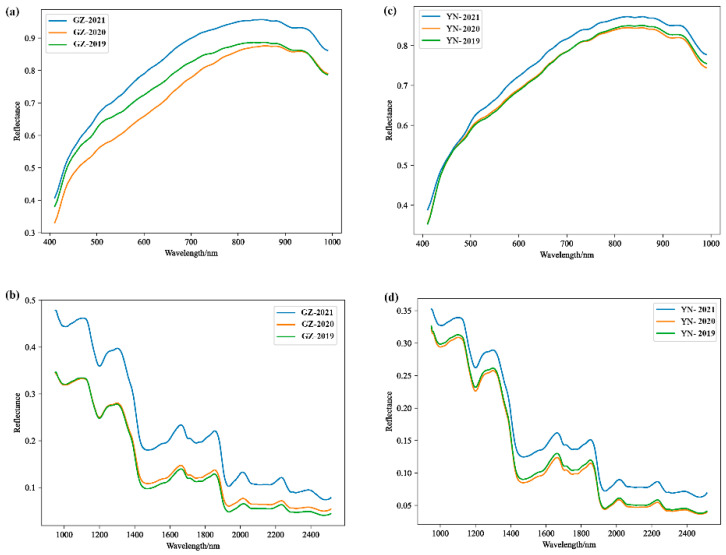
Visible to near infrared (VNIR) and short-wave infrared (SWIR) average hyperspectral features of *Coix seed* samples from different storage years. (**a**) VNIR spectra of samples from Guizhou; (**b**) SWIR spectra of samples from Guizhou; (**c**) VNIR spectra of samples from Yunnan; (**d**) SWIR spectra of samples from Yunnan.

Figure 3 illustrates the mean spectra of *Coix seed* samples collected within the VNIR spectral range (410–990 nm) and the SWIR spectral range (950–2500 nm). As depicted in Figure 3, *Coix seed* samples from various storage years in Guizhou and Yunnan exhibited analogous fluctuation patterns, with peaks and troughs appearing at comparable band positions, owing to the similar chemical composition of *Coix seed*. In addition, the average spectral reflectance of *Coix seed* from different storage years showed certain differences. It was the long-term storage that led to the changes in the internal structure and biochemical composition of *Coix seed*, thus, resulting in changes in their optical properties. For example, the peaks at 820 nm and 970 nm were caused by the N–H stretching third overtone in protein and O–H stretching second overtone in water [11]. The peaks at 1030 nm may be associated with absorption peaks of stretching vibrations of C–OH bonds in carbohydrates [41]. The valley at 1200 nm were reported to be attributed to the second overtone of the C-H stretching in carbohydrates [42]. A distinct peak at 1320 nm may be related to the double-frequency absorption bands of the C–H bonds [43]. The valleys at 1430 nm were due to the first overtones of the O-H stretching in the water absorption [44,45]. The peaks and valleys at 1860 nm, 1930 nm, 2000 nm, and 2200 nm were related to the frequency synthesis of C-H and O-H groups [46]. This further confirmed that a longer storage time would lead to the loss of nutrients in *Coix seed* and the specific spectral data could be used for identification of different storage years.

### 3.2. Classification Results

In order to achieve improved classification outcomes, this investigation employed four conventional ML methods (KNN, SVM, RF, and XGBoost) and a deep learning method ResNet to establish models to recognize *Coix seed* samples from different storage years. In this investigation, a 10-fold cross-validation approach was employed, wherein the dataset was randomly partitioned into 10 groups. One of these groups served as the test dataset, while the remaining nine groups were utilized as the training dataset to fine-tune the model’s predictive hyperparameters. Consequently, each subset was employed for testing purposes at least once.

For ML models, the model parameters were optimized through grid search, with the objective of obtaining optimal performance. For instance, in the KNN model, the number of neighbors was fine-tuned within the range of [1,10], and the weights were optimized using both ‘distance’ and ‘uniform’ options. In the RF model, the number of estimators was optimized over a range of 100 to 1000 with an interval of 100. In the SVM algorithm, three kernel functions were tested: linear, radial basis functions (RBF), and polynomial. The optimization of the parameter C ranged from 1 to 1 × 10^3^, and gamma was set to ‘scale’. For XGBoost, the learning rate was optimized within the range of [0.1, 0.01, 0.001], the number of estimators was optimized within the range of 100 to 800, and the max_depth parameter was set to [3,5,7].

**Figure 4:**
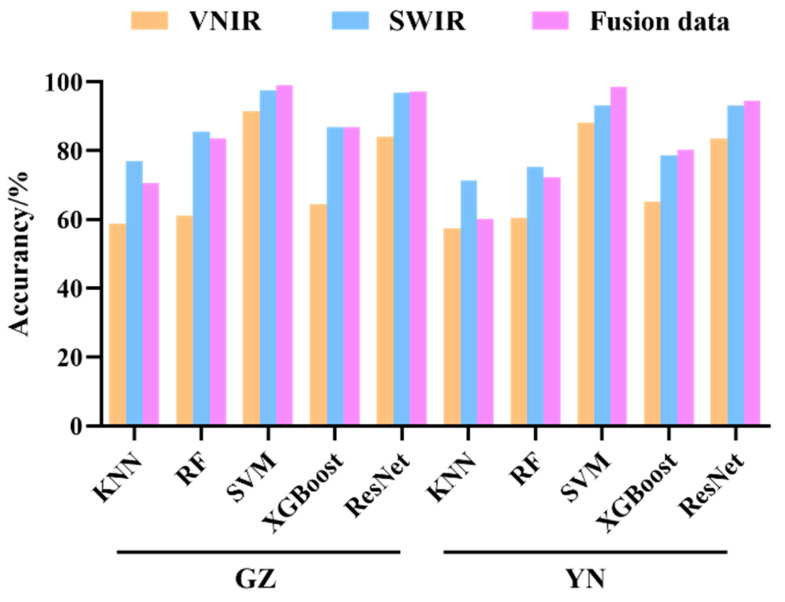
The classification accuracy of conventional ML and ResNet classifiers based on VNIR, SWIR, and fusion data.

The classification accuracy results of various ML models and the ResNet model in the VNIR and SWIR spectral ranges are presented in Table 2 and Table 3 and Figure 4, respectively. It is noteworthy that the majority of models exhibit higher accuracy in the SWIR spectral range compared to the VNIR spectral range. The classification performances of KNN, RF, and XGBoost were relatively subpar, with accuracy ranging from 57% to 87%. However, upon optimizing the parameters, the SVM models demonstrated superior classification outcomes. The accuracy of the testing set was over 88% in the VNIR range and 93% in the SWIR range for SVM models. This indicates that SVM, after fine-tuning the parameters, proved to be effective in discerning *Coix seed* samples from different storage years, with higher accuracy rates compared to other ML models.

The ResNet model was optimized using the Adam algorithm with a constant learning rate of 0.0001 and the training process was conducted for 1000 epochs. The accuracy of the ResNet model in SWIR ranges provides better classification results, with accuracy over 93%. In contrast, the classification results of the ResNet model in the VNIR spectral range were relatively lower, with an accuracy of approximately 83%.

### 3.3. Classification with VNIR and SWIR Fusion

**Figure 5:**
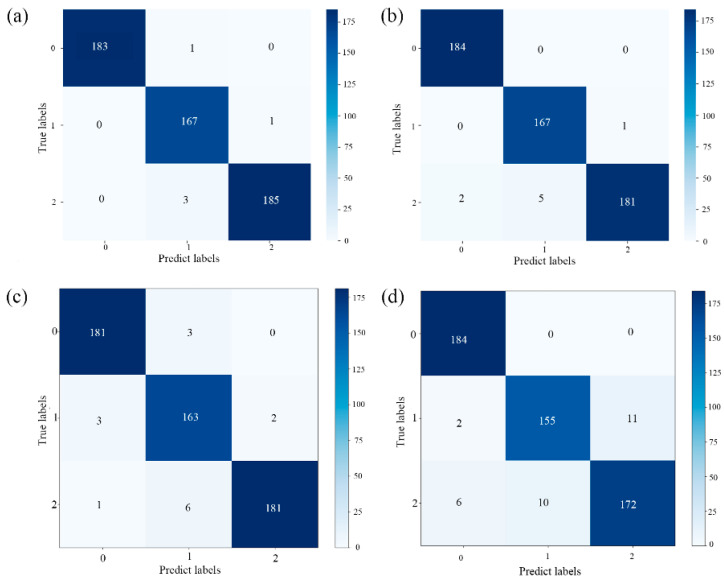
(**a**,**b**) Confusion matrix of the support vector machine (SVM) model classification results for *Coix seed* samples from Guizhou and Yunnan; (**c**,**d**) confusion matrix of the ResNet model classification results for *Coix seed* samples from Guizhou and Yunnan. 0, 1, and 2 represent samples for 2019, 2020, and 2021.

By employing a data-level fusion approach, the spectral information from the VNIR and SWIR regions of the *Coix seed* samples was merged. It is evident from Table 4 and Figure 5 that the accuracy of each model experienced a significant improvement after spectral fusion. The SVM and ResNet models achieved a classification accuracy of nearly 95% for the *Coix seed* samples from Guizhou and Yunnan provinces. This indicates the feasibility of combining HSI with both traditional ML and deep learning techniques for the classification of *Coix seed* samples from different storage years. The confusion matrix results for both models can be found in Figure 5. Subsequently, the following sections will further optimize the best spectral data preprocessing methods and feature wavelength selection for the SVM and ResNet models.

The SVM and ResNet models initially used various preprocessing techniques such as MSC, SNV, and SG to reduce spectral noise and scattering effects. The intention was to determine the most effective preprocessing method based on the results obtained. However, it is worth noting that the performance of the models constructed using these alternative preprocessing methods did not surpass that of the models built using the raw full-spectrum approach (Table 5). Therefore, we decided to continue with the SVM and ResNet modelling methods using the raw spectra for further evaluation.

### 3.4. Extraction of Spectral Feature Wavelength

Given the considerable volume of full wavelength data and the potential issues of redundancy and collinearity, it becomes necessary to employ feature wavelength selection methods. This helps in identifying the most informative data for modeling while eliminating redundant information that may interfere with the model [47]. Consequently, feature wavelength selection techniques such as CARS and SPA were utilized for further modeling and analysis. The characteristic wavelength selection results for *Coix seed* samples from Yunnan and Guizhou can be found in Supplementary Tables S1 and S2. These results provide insights into the specific wavelengths that contribute significantly to the classification of *Coix seed* samples from different storage years.

**Figure 6:**
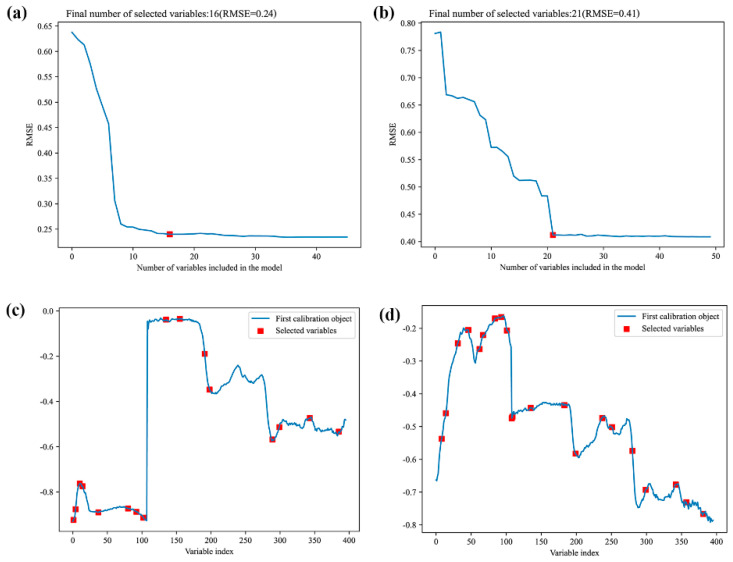
Characteristic wavelengths selection results of successive projections algorithm (SPA). (**a**,**b**) Distribution of RMSE of *Coix seed* samples from Guizhou and Yunnan; (**c**,**d**) distribution of the selected variables of *Coix seed* samples from Guizhou and Yunnan.

SPA is a forward circular variable selection algorithm. After selecting a wavelength, the maximum wavelength of the projection vector is introduced into the wavelength combination by calculating the projection on the unselected wavelength in each cycle. According to the principle that each newly selected wavelength has the least linear relationship with the previous one, the optimal combination of wavelength variables is finally selected by cyclic alternation. The quality of the model is determined by the minimum RMSE corresponding to the selected number of variables [29,30]. In the case of SPA, the wavelength variable ranged from 2 to 50, with a minimum and maximum value, respectively. Figure 6a,b shows the root-mean-square error (RMSE) change curve under different variables. For *Coix seed* samples from Guizhou and Yun-nan, the RMSE reached an optimal value after the characteristic wavelength (labelled as an open red square) was selected as 16 and 21, respectively. Figure 5c,d display the distribution of the selected characteristic wavelengths in the full spectrum band. The distribution of the retained wavelengths by SPA is presented in Supplementary Table S1.

**Figure 7:**
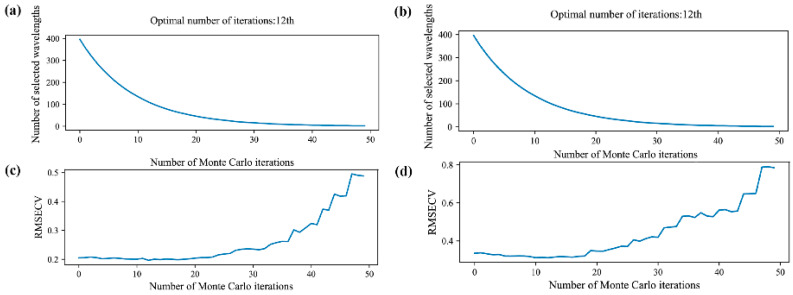
The variable selection of environmental data from competitive adaptive reweighted sampling (CARS) running. (**a**,**b**) Number of variables of *Coix seed* samples from Guizhou and Yunnan; (**c**,**d**) root-mean-square error of cross validation (RMSECV) *Coix seed* samples from Guizhou and Yunnan.

The CARS method is a feature variable selection technique that combines Monte Carlo sampling with the regression coefficient of the PLS (partial least squares) model. It identifies wavelength points with significant regression coefficients in the PLS model by employing adaptive re-weighted sampling (ARS) technology. It then eliminates wavelengths with small weights, using interactive verification to select the subset with the lowest root-mean-square error cross-validation (RMSECV) index. This approach effectively identifies the optimal combination of variables [28]. In the CARS method, 50 Monte Carlo samples were generated, and the wavelength with the smallest RMSECV was selected as the optimum using the 10-fold cross-validation method. The results of the CARS algorithm are presented in Figure 7. Figure 7a,c demonstrate that the minimum RMSECV was achieved after 12 sampling runs, with 108 retained wavelengths for the Guizhou samples. Similarly, Figure 7b,d show that the minimum RMSECV was obtained after 12 sampling runs, with 97 retained wavelengths for the Yunnan samples. The distribution of the retained wavelengths by CARS is presented in Supplementary Table S2.

### 3.5. Recognition Results of the Models on Characteristic Spectra

**Figure 8:**
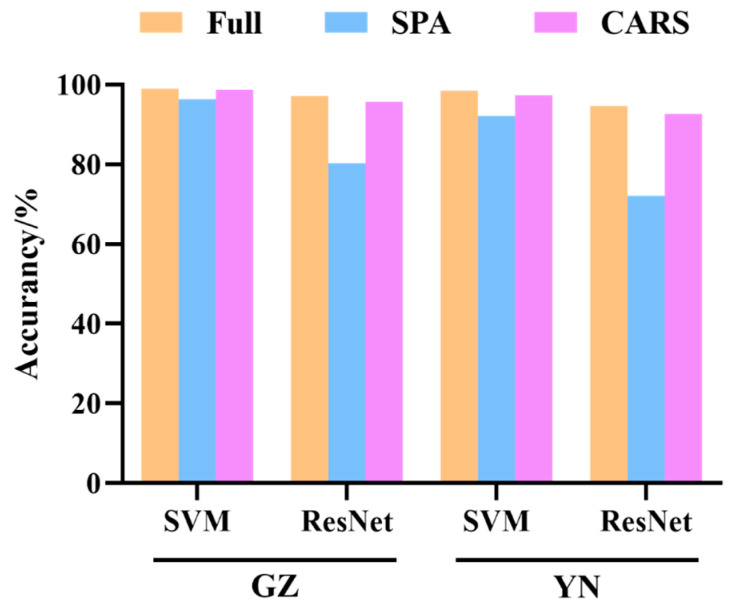
The classification accuracy of SVM and ResNet models based on characteristic spectra.

**Figure 9:**
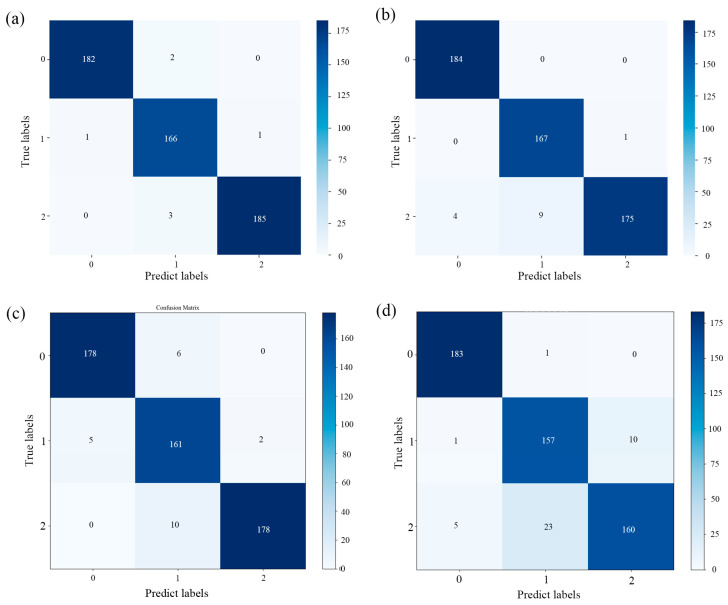
(**a**,**b**) Confusion matrix of the CARS-SVM model classification results for *Coix seed* samples from Guizhou and Yunnan; (**c**,**d**) confusion matrix of the CARS-ResNet model classification results for *Coix seed* samples from Guizhou and Yunnan. 0, 1, and 2 represent samples for 2019, 2020, and 2021.

Based on the optimal wavelengths selected by the SPA and CARS methods, SVM and ResNet models were established, and their respective performances were presented in Table 6 and Figure 8. It is evident that for the SVM model, the performance did not significantly decline after applying the SPA and CARS algorithms to select the feature bands. However, for the ResNet model, the feature bands selected by the SPA algorithm had a substantial impact on its performance. The subpar prediction results may be attributed to the deletion of some useful information during the effective wavelength selection process. Therefore, compared to the model built using the SPA method, the model constructed using the CARS method with the selected wavelengths demonstrates a significant advantage in distinguishing the storage years of *Coix seed*. In the prediction sets of Yunnan and Guizhou, the average discrimination accuracy of the CARS-SVM method was 98.05%, while the accuracy of the CARS-ResNet method was 94.15%. The confusion matrix results for the CARS-SVM and CARS-ResNet methods are depicted in Figure 9. Moreover, compared to the model constructed using the complete set of wavelengths, the accuracy of the CARS-based model only decreased by approximately 1%. This effectively showcases the efficacy of the CARS method in maintaining high precision while reducing complexity.

### 3.6. Identification and Visualization of Coix seed Samples from Different Storage Years for Validation Sets

**Figure 10:**
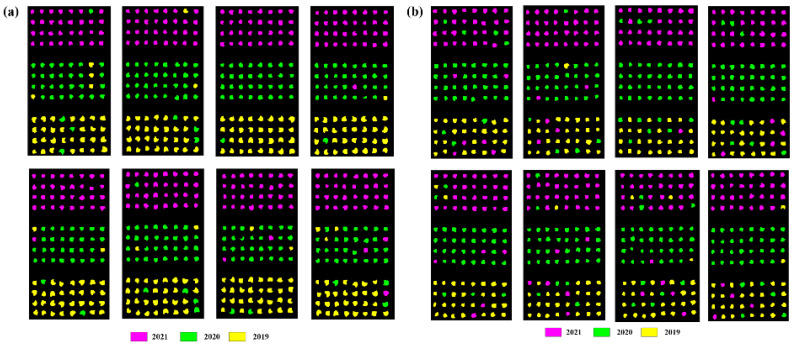
The prediction results of validation set based on the characteristic wavelength. (**a**) Guizhou; (**b**) Yunnan.

To assess the performance of the SVM and ResNet models developed using characteristic wavelength spectral data for CARS screening, the models were evaluated on external validation sets comprising 16 groups, each consisting of 96 samples. These sets represented various origins and vintages of *Coix seed*, with each vintage comprising 32 samples. The outcomes are presented in Table 7. The SVM model exhibited an average accuracy of 66.49%, while the ResNet model demonstrated an average accuracy of 87.27%. These findings suggest that conventional ML models employed for distinguishing different storage years of *Coix seed* samples are relatively ineffective. This phenomenon may be attributed to potential disparities between the external validation set and the training and testing sets, as well as the potential overfitting of the models, resulting in diminished generalization to new data and decreased accuracy. Conversely, the ResNet model, particularly designed for deep learning, proved to be more adept at discerning the different storage years of *Coix seed* samples. This observation underscores the ResNet model’s superior ability to generalize effectively when confronted with complex data. Consequently, the ResNet model, constructed utilizing characteristic wavelengths extracted from CARS, can reliably and accurately identify the storage years of *Coix seed*. Figure 10 provides a visual representation of the prediction outcomes for the validation set based on the characteristic wavelengths.

## 4. Discussion

The aging process occurs during the storage of *Coix seeds*, leading to alterations in their nutritional composition and a decline in their quality (Devaraj et al., 2020). Consequently, the determination of the storage years of *Coix seeds* holds significant importance in ensuring consumer well-being. HSI emerges as a promising non-destructive detection technique, widely applied in the assessment and classification of agricultural product quality [48].

In this study, we collected *Coix seed* samples from different storage years and employed HSI technology to apply deep learning models for their identification. The results indicate that classification models built using different spectral ranges exhibit varying performance. Overall, both traditional ML models and ResNet model achieved higher classification accuracy when based on SWIR HSI data (900–2500 nm) compared to VNIR HSI data (350–950 nm). A study on the geographical classification of *Atractylodes macrocephala* also found that the classification accuracy based on modeling in the SWIR region (93.2%) was slightly higher than that based on VNIR region (90.5%) [49]. Furthermore, data fusion enhanced the model’s precision, with average accuracy of 98.80% for the SVM model and 95.93% for the ResNet model. After optimizing the CARS feature wavelength of the data, redundant information was eliminated, reducing the number of wavelengths by approximately 70% while maintaining the accuracy of the SVM and ResNet models without compromising their performance. The overall performances of the ResNet models were found to be comparable to those of the SVM models when considering different storage years of *Coix seed*. This indicates that both support vector machines (SVM) and deep learning approaches, such as ResNet, demonstrate efficacy in processing spectral data for data analysis tasks. However, it is important to note that SVM and deep learning operate on distinct principles. SVM is a widely adopted method capable of handling both linear and nonlinear problems. In the realm of spectral data analysis, SVM with kernel functions is commonly employed, wherein the input data are mapped to higher dimensions using a kernel function and subsequently subjected to sample classification [7]. On the other hand, deep learning methods leverage multiple layers of nonlinear processing units to extract and transform features, showcasing a remarkable capability for feature learning and effective information extraction from the data [14].

Further, through the integration of ResNet models with traditional ML models for external validation, it becomes evident that the ResNet model surpasses conventional machine learning models in terms of classification accuracy. Specifically, the CARS-ResNet model achieves a recognition accuracy of 87.27% for various annual samples of *Coix seed*, surpassing the performance of CARS-SVM, which reaches 66.49%. This outcome can be attributed to the inadequate generalization ability of traditional machine learning models when applied to high-throughput data processing. When confronted with intricate data and tasks, traditional machine learning models may encounter limitations in their generalization capability, whereas methods such as deep learning and neural networks are likely to exhibit remarkable generalization prowess [50,51]. The CARS-ResNet model developed in this study enables the identification of *Coix seed* samples from different years.

The information obtained from this study holds great potential for the development of an intelligent monitoring device to assess the freshness of *Coix seed*. To further enhance its applicability and broaden its scope, future research efforts could be dedicated to developing a universal model capable of encompassing a wider range of sample types. Such an advanced monitoring device would enable real-time and accurate detection and analysis of *Coix seed* in an industrial environment, thereby improving production efficiency and quality control standards.

This paper still has several shortcomings that require further research. Firstly, in the models involved in this study, the identification of storage years of *Coix seed* relies on the integrity of the seeds. Since we used intact *Coix seed* samples to build the models, the use of powdered or fragmented samples may lead to data distortion or information loss, thereby affecting the performance of the models. Therefore, in future studies, researchers can collect more hyperspectral data from fragmented or powdered samples and apply transfer learning to improve the models’ applicability and generalization, enabling better identification of *Coix seed* samples in various forms. Secondly, relying solely on spectral characterization is insufficient to analyze the metabolomic changes during the aging process of *Coix seed*. To examine the variations in the content of each compound during *Coix seed* aging, quantitative analysis can be conducted using techniques such as UPLC-MS/MS. After identifying the key differentiating metabolites, combining spectral imaging techniques would allow regression prediction analysis.

## 5. Conclusions

This study employed HSI to extract spectral information (VNIR and SWIR) for the recognition and classification of *Coix seed* samples from different storage years. By integrating the ResNet algorithm with traditional ML methods, the spectral information of the samples was utilized to successfully identify *Coix seed* samples from Yunnan and Guizhou, encompassing three different storage years. Overall, utilizing a fusion-based modeling approach yielded higher classification accuracy compared to individual VNIR and SWIR spectral modeling. The classification accuracy of the ResNet model and SVM exceeds that of other conventional machine learning models (KNN, RF, and XGBoost). Through the application of CARS feature wavelength selection, redundant variables were further reduced, resulting in minimal impact on the model’s accuracy. When validating the model’s performance using external validation sets, the ResNet model exhibited satisfactory outcomes, achieving a recognition accuracy exceeding 85%. The comprehensive findings of this study demonstrate the successful application of HSI for the rapid and non-destructive determination of *Coix seed*’s storage vintage, presenting a novel strategy for the swift assessment of grain freshness.

## Figures and Tables

**Table 1:** The architecture of ResNet.

Layer Name	Output Size	50-Layer
Conv1	112 × 112	7 × 7, 64, stride 2 3 × 3 max pool, stride 2
Conv2_*x*	56 × 56	$\left\{\begin{array}{c} 1\times 1,\;64\\ 3\times 3,\;64\\ 1\times 1,\;256 \end{array}\right\}$
Conv3_*x*	28 × 28	$\left\{\begin{array}{c} 1\times 1,\;128\\ 3\times 3,\;128\\ 1\times 1,\;512 \end{array}\right\}$
Conv4_*x*	14 × 14	$\left\{\begin{array}{c} 1\times 1,\;256\\ 3\times 3,\;256\\ 1\times 1,\;1024 \end{array}\right\}$
Conv5_*x*	7 × 7	$\left\{\begin{array}{c} 1\times 1,\;512\\ 3\times 3,\;512\\ 1\times 1,\;2048 \end{array}\right\}$
	1 × 1	average pool, 1000-d fc, softmax

**Table 2:** The accuracy of conventional machine learning (ML) models and ResNet model for VNIR spectra.

Methods	Training Set (%)		Testing Set (%)	
	GZ	YN	GZ	YN
KNN	100.00	100.00	58.70	57.41
RF	100.00	100.00	61.30	60.37
SVM	93.49	89.05	91.48	88.33
XGBoost	100.00	100.00	64.44	65.19
ResNet	100.00	100.00	84.07	83.52

**Table 3:** The accuracy of conventional ML models and ResNet model for SWIR spectra.

Methods	Training Set (%)		Testing Set (%)	
	GZ	YN	GZ	YN
KNN	100.00	100.00	76.85	71.48
RF	100.00	100.00	85.55	75.18
SVM	98.65	95.79	97.59	93.33
XGBoost	100.00	99.92	86.85	78.52
ResNet	100.00	100.00	96.85	93.15

**Table 4:** The accuracy of conventional ML models and ResNet model for data-level information fusion.

Methods	Training Set (%)		Testing Set (%)	
	GZ	YN	GZ	YN
KNN	100.00	100.00	70.56	60.00
RF	100.00	100.00	83.52	72.22
SVM	99.84	99.23	99.07	98.52
XGBoost	100.00	100.00	86.85	80.18
ResNet	100.00	100.00	97.22	94.63

**Table 5:** Performance of established classification models developed by full spectra with preprocessing.

Pretreatment	Methods	Training Set (%)		Testing Set (%)	
		GZ	YN	GZ	YN
MSC	SVM	99.60	99.60	99.25	99.26
	ResNet	100.00	100.00	95.37	93.15
SNV	SVM	100.00	99.37	99.07	98.70
	ResNet	100.00	100.00	94.44	92.04
SG	SVM	99.13	99.13	98.52	98.52
	ResNet	100.00	100.00	97.03	94.81

**Table 6:** The optimal SVM and ResNet modeling results developed by selected wavelengths based on different methods.

Data Type	Methods	Training Set (%)		Testing Set (%)	
		GZ	YN	GZ	YN
SPA	SVM	98.02	93.10	96.30	92.22
	ResNet	100.00	100.00	80.37	72.22
CARS	SVM	99.12	98.49	98.70	97.40
	ResNet	100.00	100.00	95.70	92.59

**Table 7:** Identification results of *Coix seed* samples of different years in the external validation set.

Methods	Group	Correct Number		Accuracy (%)	
		GZ (0/1/2)	YN (0/1/2)	GZ	YN
CARS-SVM	1	34/25/37	32/43/21	50.00	77.08
	2	34/30/32	29/38/29	59.38	80.21
	3	31/27/38	37/39/20	60.42	70.83
	4	32/33/31	38/37/21	57.29	69.79
	5	38/24/34	33/41/22	61.46	78.13
	6	35/24/37	36/37/23	57.29	82.29
	7	36/27/33	34/39/23	48.96	76.04
	8	42/21/33	30/36/30	59.38	72.92
	Mean acc	-	-	56.77	75.91
CARS-ResNet	1	33/32/31	21/30/45	91.67	87.5
	2	25/34/37	24/42/30	93.75	88.54
	3	31/33/32	23/37/36	98.96	86.46
	4	31/32/33	29/35/32	97.92	86.46
	5	32/31/33	25/36/35	94.79	86.46
	6	30/35/31	26/38/32	92.71	83.33
	7	32/30/34	24/37/35	93.75	87.5
	8	31/31/34	21/30/45	89.58	87.5
	Mean acc	-	-	94.14	86.72

## Data Availability

The original contributions presented in the study are included in the article, further inquiries can be directed to the corresponding author.

## Supplementary Materials

The following supporting information can be downloaded at: https://www.mdpi.com/article/10.3390/foods13030498/s1, Table S1: The feature wavelengths selected by successive projections algorithm; Table S2: The feature wavelengths selected by competitive adaptive reweighted sampling algorithm.
