# Supplementary material for: Identification and Classification of Coix seed Storage Years Based on Hyperspectral Imaging Technology Combined with Deep Learning
Source: Foods. 2024 Feb 4;13(3):498. doi: 10.3390/foods13030498 (PMC10855119; doi:10.3390/foods13030498)
Supplement: Supplementary file 1 [file foods-13-00498-s001.zip › foods-2804908-supplementary.pdf]

Identification and classification of *Coix seed* storage years based on hyperspectral imaging technology combined with deep learning

Ruibin Bai[a], Junhui Zhou[a], Siman Wang[a], Yue Zhang[a], Tiegui Nan[a], Chu Zhang[b]*, Jian Yang[a]*

[a] State Key Laboratory for Quality Ensurance and Sustainable Use of Dao-di Herbs, National Resource Center for Chinese Materia Medica, China Academy of Chinese Medical Sciences, Beijing, 100700, China

[b] School of Information Engineering, Huzhou University, Huzhou, 313000, China

Table S1 The feature wavelengths selected by successive projections algorithm.

	Number	Characteristic wavelengths (nm)
YN	21	1651.82, 773.39, 1728.12, 453.75, 914.25, 1886.18, 957.59, 865.49, 578.36, 1095.88, 1444.70, 746.30, 2224.10, 486.26, 2305.86, 1357.49, 659.62, 948.72, 1989.74, 954.17, 2436.67
GZ	16	1401.10, 1204.89, 908.84, 2229.56, 1439.25, 963.01, 843.82, 1095.88, 1989.74, 486.26, 464.59, 610.86, 432.079969, 2458.47, 1935.23, 415.83

Table S2 The feature wavelengths selected by competitive adaptive reweighted sampling algorithm.

	Number	Characteristic wavelengths (nm)
YN	108	426.66, 448.33, 453.75, 464.59, 470.00, 486.26, 491.67, 535.02, 540.43, 551.27, 556.69, 600.03, 605.44, 670.46, 692.13, 702.96, 773.39, 784.23, 789.64, 795.06, 800.48, 827.57, 832.98, 838.40, 843.82, 865.49, 908.83, 914.25, 919.67, 973.84, 979.26, 984.68, 948.72, 954.17, 959.62, 965.07, 1003.22, 1019.57, 1025.02, 1030.47, 1035.92, 1041.37, 1068.63, 1074.08, 1139.48, 1144.93, 1150.38, 1155.83, 1161.28, 1188.53, 1204.89, 1215.79, 1221.24, 1226.69, 1232.14, 1237.59, 1243.04, 1248.49, 1302.99, 1308.44, 1313.89, 1319.34, 1330.24, 1335.69, 1341.14, 1346.60, 1422.90, 1428.35, 1450.15, 1461.05, 1466.50, 1471.95, 1510.11, 1531.91, 1537.36, 1597.31, 1602.76, 1608.21, 1613.66, 1624.56, 1630.01, 1640.91, 1673.62, 1706.32, 1711.77, 1728.12, 1793.52, 1798.97, 1809.88, 1858.93, 1864.38, 1869.83, 1897.08, 1913.43, 1929.78,

		1967.94, 1978.84, 1984.29, 2016.99, 2066.04, 2098.74, 2196.85, 2240.45, 2267.71, 2300.41, 2311.31, 2327.66, 2507.52
GZ	108	464.59, 486.26, 507.93, 513.34, 535.02, 540.43, 545.85, 551.27, 567.52, 589.19, 610.86, 621.70, 627.11, 643.37, 665.04, 670.46, 719.21, 724.63, 730.05, 735.47, 746.30, 784.23, 800.48, 822.15, 865.49, 870.91, 876.33, 887.16, 892.58, 903.41, 914.25, 946.76, 957.59, 968.43, 954.17, 959.62, 992.32, 1025.02, 1074.08, 1095.88, 1150.38, 1166.73, 1215.79, 1226.69, 1237.59, 1243.04, 1248.49, 1270.29, 1275.74, 1281.19, 1292.09, 1308.44, 1319.34, 1324.79, 1330.24, 1335.69, 1341.14, 1357.50, 1362.95, 1379.30, 1390.20, 1395.65, 1401.10, 1406.55, 1412.00, 1428.35, 526.46, 1575.51, 1580.96, 1586.41, 1635.46, 1662.72, 1679.07, 1684.52, 1695.42, 1733.57, 1837.13, 1864.38, 1875.28, 1880.73, 1886.18, 1891.63, 1902.53, 1973.39, 1978.84, 1984.29, 2006.09, 2011.54, 2027.89, 2033.34, 2055.14, 2066.04, 2109.65, 2115.10, 2120.55, 2131.45, 2175.05, 2185.95, 2218.65, 2224.10, 2229.55, 2240.45, 2256.81, 2278.61, 2365.81, 2442.12, 2458.47, 2480.27
